# Comparison of DNA purification methods for high‐throughput sequencing of fungal communities from wine fermentation

**DOI:** 10.1002/mbo3.1321

**Published:** 2022-10-25

**Authors:** Antoine Gobert, Marie Sarah Evers, Christophe Morge, Céline Sparrow, Vincent Delafont

**Affiliations:** ^1^ SAS Sofralab Magenta France; ^2^ Laboratoire Ecologie et Biologie des Interactions, Equipe, Microorganismes, Hôtes, Environnements, Université de Poitiers UMR CNRS 7267 Poitiers France

**Keywords:** DNA, fermentation, fungal diversity, metagenomic, wine, yeast

## Abstract

High‐throughput sequencing approaches, which target a taxonomically discriminant locus, allow for in‐depth insight into microbial communities’ compositions. Although microorganisms are historically investigated by cultivation on artificial culture media, this method presents strong limitations, since only a limited proportion of microorganisms can be grown in vitro. This pitfall appears even more limiting in enological and winemaking processes, during which a wide range of molds, yeasts, and bacteria are observed at the different stages of the fermentation course. Such an understanding of those dynamic communities and how they impact wine quality therefore stands as a major challenge for the future of enology. As of now, although high‐throughput sequencing has already allowed for the investigation of fungal communities, there is no available comparative study focusing on the performance of microbial deoxyribonucleic acid (DNA) extraction in enological matrixes. This study aims to provide a comparison of five selected extraction methods, assayed on both must and fermenting must, as well as on finished wine. These procedures were evaluated according to their extraction yields, the purity of their extracted DNA, and the robustness of downstream molecular analyses, including polymerase chain reaction and high‐throughput sequencing of fungal communities. Altogether, two out of the five assessed microbial DNA extraction methods (DNeasy PowerSoil Pro Kit and E.Z.N.A.® Food DNA Kit) appeared suitable for robust evaluations of the microbial communities in wine samples. Consequently, this study provides robust tools for facilitated upcoming studies to further investigate microbial communities during winemaking using high‐throughput sequencing.

## Graphical abstract

This study aims at exploring the most suitable experimental procedure for allowing the high‐throughput sequencing analysis of microbial communities in wine samples.

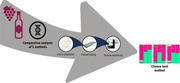

## 1 INTRODUCTION

Vineyard and grape microbial diversity have long been studied using microbiological methods, relying on agar plate cultivation, microscopy, and biochemical characterization (König et al., 2017). However, such techniques, implying a mandatory microbial cultivation step, exclude uncultivable wine micro‐organisms from downstream analyses (Amann et al., 1995; Curtis et al., 2002). Polymerase chain reaction (PCR)‐based methods with deoxyribonucleic acid (DNA) extracted directly from the environment, allow microbial investigation relying on the presence of nucleic acids for detection and identification rather than their requirements and growth capacities on given media (Morgan et al., 2017). Such culture‐independent methods appear to often be more sensitive, faster, and characterized by a higher accuracy than culture‐dependent ones (Lv et al., 2013).

High‐throughput sequencing (HTS), either relying on an untargeted shotgun sequencing approach (i.e., metagenomics) or targeted on a specific locus (i.e., amplicon sequencing), stands as a deeply interesting, cultivation‐free concept for studying microbial biodiversity (Handelsman et al., 2018; He et al., 2007; Riesenfeld et al., 2004). Such methods can give access to the entirety of the genetic resources present in an environment by directly isolating and amplifying DNA from it (Handelsman et al., 2018). Therefore, HTS (metagenomics and amplicon sequencing) has begun to stand as a relevant method to investigate microbial composition during winemaking (Bokulich et al., 2016; Stefanini & Cavalieri, 2018; Sternes et al., 2017; Zepeda‐Mendoza et al., 2018). However, wine products are complex matrices, containing large amounts of PCR inhibitors, such as polyphenols and polysaccharides (Işçi et al., 2014), therefore rendering such molecular analyses difficult in their execution. As a consequence, the choice of DNA extraction method is paramount in wine microbial diversity assessments, since it has to be isolated in sufficient quality and quantity to envision HTS‐based analyses (Işçi et al., 2014). In enology, DNA isolation techniques are generally used for genetic varietal authentication of wine (Baleiras‐Couto & Eiras‐Dias, 2006; Barrias et al., 2019; Catalano et al., 2016; Işçi et al., 2014; Pereira et al., 2011; Siret et al., 2000). Few comparative studies are available about DNA extraction adapted for downstream analyses of microbial diversity, especially for yeasts (Hall et al., 2019; Jara et al., 2008). Thus, HTS approaches provide a pertinent and timely tool for a better understanding of the impact of these practices on yeast diversity. This is particularly relevant in assessing the impact of biological alternatives (Roudil et al., 2019; Simonin et al., 2018) or bio‐sourced chemicals (Bağder Elmacı et al., 2015; Castro Marín et al., 2019; Taillandier et al., 2015).

To our knowledge, there is presently a lack of comparative studies assessing the efficacy and applicability of various DNA extraction methods optimized for HTS in enological conditions. This currently hampers the robust and repeatable study of microbial communities in such complex matrices. Thus, the present study aims to assess various readily available methods for extracting high‐quality DNA, allowing for HTS of fungal internal transcribed spacer (ITS) to characterize the fungal diversity at three fermentation stages (must, mid‐fermentation, and end fermentation). For this, four commercial kits specifically designed for extraction of DNA from foods or soil are compared. A conventional extraction using cetyltrimethylammonium bromide–polyvinylpyrrolidone (CTAB‐PVP) buffer and phenol/chloroform (PC) solution was chosen to evaluate the kit performance based on the DNA yields and quality. The PCR amplification success was evaluated, and high throughput amplicon sequencing of fungal communities was conducted and compared for all validated methods.

## 2 MATERIALS AND METHODS

### 2.1 Microorganism and matrix

A commercial strain of *Saccharomyces cerevisiae* “Italica CR1” (Sofralab) was used for the inoculation of the must. The matrix used in this study is a thermic‐macerated Syrah must, which comes from the Canton de Montagnac area, region of Languedoc (South of France), harvested in 2020.

### 2.2 Fermentation conditions

After the selection of berries only, the crushed grapes were treated with 3 g/hL of sulfur dioxide and then pooled in a stopover tank for an estimated time of approximately 60 min. Crushed grapes were then continuously withdrawn by a Monho pump to pass through a tube heat exchanger, which was fed with hot water at 93°C produced by a boiler, through which the product passes 18 times within 2 min. The crushed grapes entered the exchanger at a temperature of about 25°C–27°C and exited at 80°C. A second stopover tank was filled with the hot must and a maceration step of 15 h was applied. At the end of the maceration, the temperature was decreased to 50°C. The must was squeezed, and a flotation process was carried out to reach a turbidity of about 500 NTU. The treated must was placed in a fermentation tank at 25°C and 10 L were sampled in a 20 L glass jar. Then the temperature was maintained at 20°C during the experiment. No extra addition of sulfur dioxide was applied at the beginning of the fermentation to favor the development of non‐*Saccharomyces* yeasts. After 48 h, *S. cerevisiae* “Italica CR1” (Sofralab) was inoculated with an initial cell population of 1.0 × 10^6^ cells/mL to ensure its implementation after rehydration, as recommended by the manufacturer. During the process, three samplings were carried out. First in the must, then at mid fermentation, and at the end of the fermentation (Table 1). Each sample was centrifuged at 10,000*g* for 5 min and the supernatant was used for analysis. Total sugar, ethanol, volatile acidity concentrations, and pH were determined by FTIR spectroscopy with an OenoFoss (FOSS Electric).

**Table 1:** Enological parameters for each step of the fermentation sampling to evaluate the DNA extraction performance

Parameters	Must	Mid fermentation	End fermentation
Sugars (g/L)	220	112	0.8
Ethanol (% v/v)	ND	6.3	12.9
Volatile acidity (g/L)	ND	0.13	0.21
pH	3.49	3.5	3.52

Abbreviation: ND, not detected.

### 2.3 DNA extraction and purification

**Figure 1:**
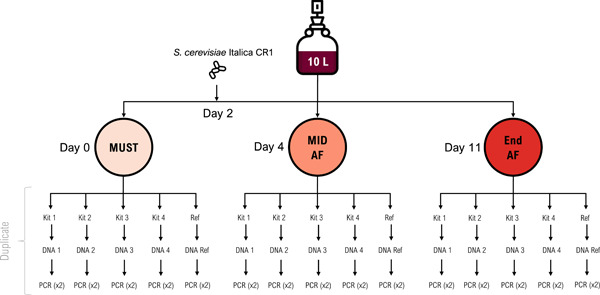
Overview of the workflow used in the study. From 10 L of thermic macerated Syrah must, three samplings were carried out. First at day 0 (must), after 4 days (mid fermentation), and after 11 days (end fermentation). *Saccharomyces cerevisiae* Italica CR1 was inoculated on day 2. The extraction and purification of DNA were performed using the four commercial kits and the reference (Jara et al., 2008) in duplicate. Kit 1: E.Z.N.A.® Food DNA, Kit 2: DNeasy PowerSoil Pro, Kit 3: DNeasy Mericon Food, and Kit 4: VINEO™ Extract DNA, Ref: CTAB‐PVP‐PC. AF, alcoholic fermentation.

Four commercial kits for DNA extraction were selected, mainly based on their use in the literature for purposes like ours, to evaluate the best technical route. The selection was composed of E.Z.N.A.® Food DNA Kit (Omega Bio‐Tek), DNeasy PowerSoil Pro Kit (Qiagen), DNeasy Mericon Food Kit (Qiagen), and VINEO™ Extract DNA Kit (Bio‐Rad Laboratories Inc.). The CTAB‐PVP‐PC extraction method was used as a reference (Jara et al., 2008). Ultimately, five different methods were compared in duplicate (Figure 1).

For all samples, 50 mL of must, must under fermentation, or wine was centrifuged at 10,000*g* for 10 min. Then, 200 mg of the pellet (wet matter) was used for the extraction of DNA. For each kit, the extraction and purification of the DNA were carried out according to the manufacturer's instructions. The CTAB‐PVP‐PC protocol is based on the work of Jara et al. (2008). The DNA samples were eluted in 100 µL of ddH_2_O and maintained at −20°C until use. The determination of the samples' purity and quantity were based on measurements performed using a Nanodrop^TM^ One Spectrophotometer (Thermo Scientific).

### 2.4 PCR conditions

To amplify the fungal DNA from samples at different stages of fermentation, fungal ITS loci were amplified using BITS (5′ CTACCTGCGGARGGATCA 3′) and B58S3 (5′ GAGATCCRTTGYTRAAAGTT 3′) primers (Bokulich & Mills, 2013). Note, PCR was performed in a 50 µL reaction volume containing Q5 buffer 5× (2 mM Mg^2+^ final concentration), 500 nM of each primer, 200 µM of each dNTPs, and 1 U of Q5 High Fidelity DNA Polymerase (New England Biolabs). Genomic DNA (25 ng) was used as a template. The PCR cycling consisted of an initial denaturation at 98°C for 2 min, followed by 30 cycles of 98°C for 10 s, 55°C for 30 s, 72°C for 60 s, and a final extension of 72°C for 2 min. All amplifications were performed in a T100 Touch Thermal Cycler (Bio‐Rad Laboratories Inc.).

### 2.5 Electrophoresis

Horizontal electrophoresis (Bio‐Rad) was used to analyze the PCR products using 2% (w/v) agarose gel with Midori Green Advance (Nippon Genetics), in 0.5× TBE buffer. For each sample, 5 µL of PCR product plus 1 µL of loading dye (Promega) were placed in wells. For electrophoresis, a migration at 100 V for 40 min was performed. The gel was exposed to ultraviolet light, and the picture was taken with a gel documentation system (Chemidoc gel touch; Bio‐Rad).

### 2.6 ITS‐amplicon preparation and sequencing

Amplicon replicates (where amplification of the ITS region was obtained) were pooled and purified using MinElute PCR Purification Kit (Qiagen). The concentration of DNA was adjusted to 50 ng/µL. Libraries were prepared using the NEBNext® Ultra II DNA Library Prep Kit for Illumina (NewEngland Biolabs). Sequencing was performed on the Illumina MiSeq sequencing platform with the V3 Illumina chemistry (2 × 150 cycles) at the ICM Institute (https://icm-institute.org/fr/) according to standard protocols.

### 2.7 Bioinformatics analyses

Demultiplexed Fastq files were imported and processed using the QIIME2 software package (Bolyen et al., 2019). Quality control, which consists of sorting out low quality (quality score <30) and chimeric sequences, as well as merging of paired reads, was performed using DADA2 with standard parameters, as implemented in the QIIME2 (Bolyen et al., 2019; Callahan et al., 2016; Table A1). Amplicon sequence variants (ASV) were identified based on this curated data set, using DADA2. The resulting ASV were further used for taxonomic affiliation, using the UNITE ITS database v 8.2 along with the VSEARCH algorithm (Abarenkov et al., 2020; Rognes et al., 2016).

## 3 RESULTS AND DISCUSSION

### 3.1 Evaluation of kit performance for DNA extraction

For this study, a set of three sample categories was produced. First in the must (immediately after extraction of the juice), then at mid fermentation (when half of the sugars were consumed), and at the end of the fermentation (<2 g/L of sugars) (Table 1). From these samples, four commercial kits for DNA extraction were selected, mainly based on their use in the literature for purposes like ours, to evaluate the best technical route. The selection was composed of E.Z.N.A.® Food DNA Kit (Omega Bio‐Tek), DNeasy PowerSoil Pro Kit (Qiagen), DNeasy Mericon Food Kit (Qiagen) and VINEO™ Extract DNA Kit (Bio‐Rad Laboratories Inc.). Those kits were selected because they comprise specific steps for mechanical lysis (DNeasy PowerSoil Pro), optimized chemical lysis (E.Z.N.A.® Food DNA Kit), dealing with an inhibitor in complex matrices (DNeasy PowerSoil Pro). Other methods were specifically developed for food and beverage analyses (DNeasy Mericon Food, VINEO^TM^). The cetyltrimethylammonium bromide—polyvinylpyrrolidone‐ phenol‐chloroform extraction method (CTAB‐PVP‐PC) was used as a reference (Jara et al., 2008).

**Figure 2:**
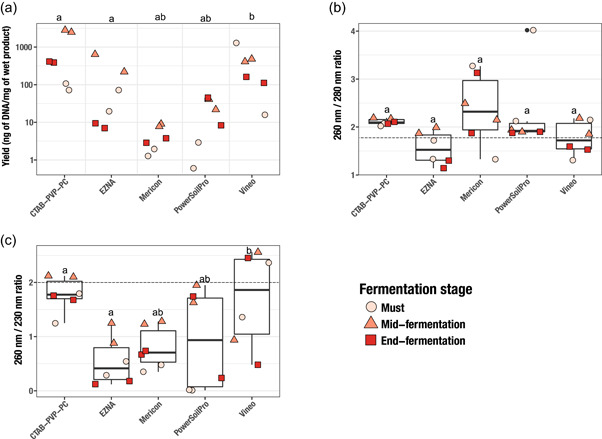
Yield and purity ratio of DNA extracted from wine samples at three different stages of fermentation. For each method, the experiments were performed in duplicate and all values (must, mid fermentation, and end fermentation) were considered for statistical analyses (*n* = 6). Shapiro–Wilk test was used to evaluate the normal distribution of data. Differing letters above the box plot indicate significance at a *p* value of <0.05 using the Kruskal–Wallis test. Thick lines in boxplots indicate the median value. The dashed line indicates ideal, targeted values for absorbance ratios.

**Figure A1:**
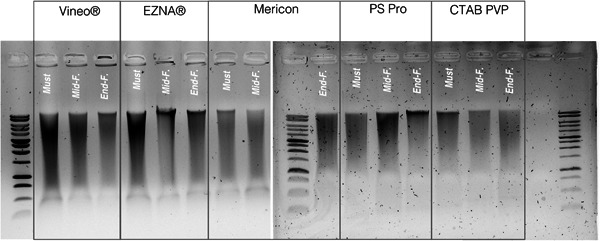
Gel electrophoresis of whole DNA extracts. Horizontal electrophoresis was performed on whole extracted DNA from all tested extraction methods. Migration was achieved in a 0.9% agarose gel, in 0.5× TBE buffer. For each sample, 50 ng of DNA was deposited and migrated for 30 min under a constant voltage of 100 V. The gels were exposed to ultraviolet light, and the picture was taken with a gel documentation system (Chemidoc gel touch; Bio‐Rad). The ladder used in the presented gels is the 1 kb DNA ladder (Promega).

Whole DNA extracts were subjected to gel migration, showing overall a comparable quality across the tested methods (Figure A1). The best yield was obtained with the CTAB‐PVP‐PC method and the Vineo^TM^ kit, with values ranging from 71 to 2800 ng of DNA/mg of wet product independently of the winemaking steps. Then, EZNA® and PowerSoil Pro kits showed intermediate values ranging from 7 to 638 ng of DNA/mg of wet product. The Mericon Food kit is the kit showing the lowest rate with yields ranging from 1 to 7.7 ng of DNA/mg of wet product. Interestingly, samples from mid fermentation gave the best yields, regardless of the DNA extraction method (Figure 2a). This result could be explained by the high concentration of yeasts (especially *S. cerevisiae*) compared to the must and the limited concentration of metabolites such as tannins found at the end of the fermentation, which may limit DNA extraction performance (Garrido & Borges, 2013), while ethanol concentration remains low at this stage.

Additionally, as an indicator of sample purity, the ratios of absorbance values at 260 versus 280 nm (A260/A280) and at 260 versus 230 nm (A260/A230) were determined. A 260/280 ratio of ~1.8 is generally accepted as “pure” for DNA (Wilfinger et al., 1997). Usually, protein contamination has a strong effect on the A260/A230 and a small effect on the A260/A280. So, if the A260/A280 reflects signs of protein contamination, relatively large amounts of proteins are present. Additionally, this implies that at low DNA concentrations, protein contamination has a large effect on purity ratios, but at high‐DNA concentrations, it may be hardly detectable (Olson & Morrow, 2012).

Our results (Figure 2b) did not show a difference in A260/A280 ratios between the methods. So based on this ratio, the DNA purity was considered acceptable (~2) and similar, regardless of the method used. However, considering the low DNA concentrations in the Mericon kit, the larger distribution of data could be explained by the strong impact of protein contamination and/or RNA co‐extraction (not necessarily more important than in the other conditions where the DNA concentration is higher). Finally, the A260/A230 is a sensitive indicator of contaminants that absorb at 230 nm. These contaminants are significantly more numerous than those absorbing at 280 nm and include chaotropic salts such as guanidine thiocyanate and guanidine hydrochloride, EDTA, nonionic detergents, phenol, and proteins. For this ratio, a value of ~2.2 is considered acceptable for amplification application. Overall, all extraction methods showed A260/A230 ratio under 2.2 which indicates the presence of contaminants and most likely proteins. Particularly for EZNA®, Mericon, and PowerSoil Pro kits, where the mean ratio ranged from 0.54 to 0.93. As the effect of protein contamination on purity ratios, A260/A230 is also dependent on DNA concentration, the contaminations in the CTAB‐PVP‐PC method and the Vineo^TM^ kit could be underestimated. Similar yields and ratio results were obtained for DNA isolation during winemaking in the context of wine traceability (Catalano et al., 2016).

### 3.2 PCR inhibition assay

**Figure A2:**
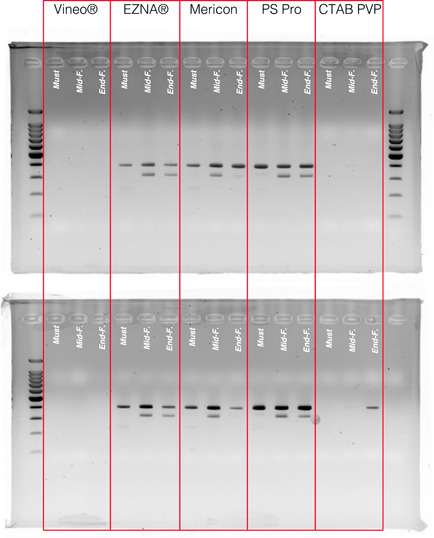
Gel electrophoresis of DNA amplification product. (a) Replicate 1, (b) Replicate 2. Horizontal electrophoresis (Bio‐Rad) was used to analyze the PCR products using 2% (w/v) agarose gel with Midori Green Advance (Nippon Genetics), in 0.5× TBE buffer. For each sample, 5 µL of PCR product plus 1 µL of loading dye (Promega) were placed in wells. For electrophoresis, a migration at 100 V for 40 min was performed. The gel was exposed to ultraviolet light, and the picture was taken with a gel documentation system (Chemidoc gel touch; Bio‐Rad). The ladder used in the presented gels is the 100 bp DNA ladder (Promega).

To determine whether PCR inhibitors were present in the purified DNA extracts, the internal transcribed spacer ITS1‐5.8S rRNA‐ITS2 region was amplified using the primer set BITS/B58S3, which is expected to yield an amplicon of approximately 300 nucleotides, depending on the fungal species present (Bokulich & Mills, 2013). Of the five methods, only E.Z.N.A.® Food DNA, DNeasy PowerSoil Pro Kit, and DNeasy Mericon Food Kit allowed repeatable amplification of all DNA extracts, even though the CTAB‐PVP‐PC method and the Vineo^TM^ kit have the best yield extraction performance (Table 2; Figure A2). Amplification issues with these two methods were not solved by modifying the amount of DNA matrix (from 1 to 100 ng) in PCR reactions. In wine samples, the presence of phenolic compounds and polysaccharides is the main cause of failure to amplify during PCR. Removing these molecules was intensively studied but mainly to purify plant DNA (Aboul‐Maaty & Oraby, 2019; Fang et al., 1992; Marsal et al., 2011, 2013; Pereira et al., 2011; Sahu et al., 2012). Few pieces of data are available about the impact of the matrix to purify and amplify microorganism DNA (Longin et al., 2016; Tessonnière et al., 2009). From these previous studies, it seems that the addition of insoluble polyvinylpolypyrrolidone (PVPP) at 1% (v/v) during bacterial DNA extraction succeeded in eliminating PCR inhibitors from red wine. In our study, a similar nonreticulated form of polyvinylpyrrolidone (PVP) was used, based on an established protocol (Cold Spring Harbor Protocols, 2009). Unfortunately, the use of this procedure did not appear to be repeatably satisfactory in the present work. Similar results were observed in other studies (red wine and vinegar; Jara et al., 2008) focused on bacterial DNA. Confronted with purity ratios, the CTAB‐PVP‐PC method and the Vineo^TM^ kit showed satisfactory results, but the large quantity of DNA might mask a significant proportion of PCR inhibitors.

**Table 2:** Amplification of the ITS region using amplicons from the different DNA extraction methods

Extraction methods of DNA	Must	Mid fermentation	End fermentation
E.Z.N.A.® Food DNA Kit	+	+	+
DNeasy PowerSoil Pro Kit	+	+	+
DNeasy Mericon Food Kit	+	+	+
VINEO™ Extract DNA Kit	−	−	−
CTAB‐PVP‐PC	−	−	−

*Note*: The experiments were performed in technical and biological duplicates.

+, Repeatable signal detected on agarose gel; −, no repeatable signal detected.

### 3.3 HTS of fungal diversity

**Figure A3:**
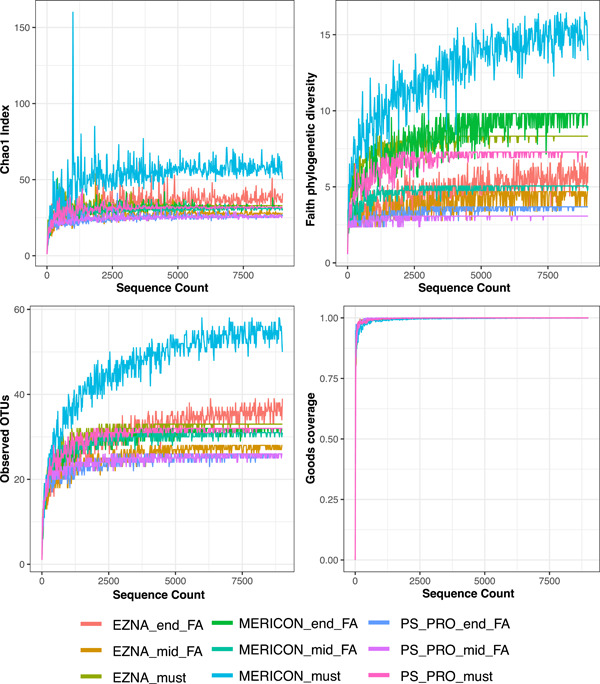
Rarefaction analyses indicate that sequencing depth was sufficient to cover the full taxonomic richness within all samples. Rarefaction indices were calculated using QIIME2, and curves were generated in the R environment using dplyr and ggplot2.

The PCR amplicons obtained by using the three kits (i.e., E.Z.N.A.® Food DNA, DNeasy PowerSoil Pro Kit, and DNeasy Mericon Food Kit; see Figure A2) allowing for a repeatable amplification of all samples were used for HTS (Illumina MiSeq. 2 × 150 cycles) of fungal communities in samples, representing nine samples (duplicates were pooled before sequencing). Overall, a total of 410, 573 raw sequences were generated from a single sequencing run (freely accessible under project number PRJNA746771 at the Sequence Read Archive). Quality control, including sorting out of low quality and chimeric sequences, was performed using DADA2, implemented in the QIIME2 software package, leaving a total of 285'862 high‐quality sequences (Bolyen et al., 2019; Callahan et al., 2016; Table A1). Rarefaction analyses suggested that sequencing depth was largely sufficient for describing community composition (Figure A3). Sequence diversity within samples was assessed by the identification of amplicon sequence variants (ASV). Overall, 26–59 ASV were identified in the analyzed samples, which were classified in 4–14 taxa (Table 3).

**Table 3:** Sequences features of samples, according to the extraction method and fermentation stage

	E.Z.N.A® Food DNA			DNeasy Mericon			DNeasy PowerSoil Pro		
	Must	Mid fermentation	End fermentation	Must	Mid fermentation	End fermentation	Must	Mid fermentation	End fermentation
No. of sequences	9235	30,042	86,569	42,449	27,018	22,730	16,430	24,912	26,476
Number of ASV	33	28	40	59	31	32	32	26	26
Number of identified taxa	10	9	5	14	5	8	9	4	4
Shannon's *H*	2.329	2.109	2.106	2.037	2.316	1.865	2.28	2.282	2.162
Chao‐1	33	28	40	59	31	32	32	26	26

*Notes*: Chao‐1 index represents a richness indicator, directly related to the number of detected ASV. Shannon's *H*‐diversity index.

Overall, only minor variations were observed by comparing all samples for their diversity. No significant differences in numbers of identified ASV or species were observed, by comparing the three extractions kits (Kruskal–Wallis test, *p* > 0.1). A stage‐dependent effect might be observed, suggesting a decrease in the number of identified taxa along with the fermentation process (Kruskal–Wallis test, *p* = 0.08244). In accordance, between 9 and 14 taxa could be identified in must samples, compared with 4–8 taxa identified in end fermentation samples (Table 3).

**Figure 3:**
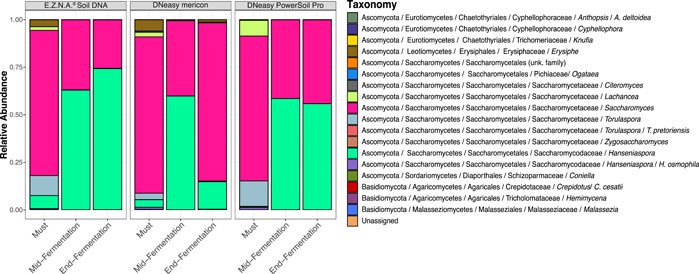
Fungal diversity identified in samples, according to the fermentation stage and the extraction method. Relative abundances of identified taxa were collected using QIIME2, extracted, and visualized in an R environment using ggplot2 (Wickham, 2016).

Taxonomic affiliations of ASV were achieved by confronting sequences against the UNITE database, using the VSEARCH algorithm (Abarenkov et al., 2020; Rognes et al., 2016). Hence, a taxonomic profile could be determined, according to the extraction method and the sample stage (Figure 3). Sequences affiliated with *Saccharomyces* were highly abundant in all samples, at all stages, though their highest relative abundance could be observed in the must stage (before inoculation of the commercial strain). Detection of *Saccharomyces* in mid fermentation and end fermentation is logical and corresponds to the inoculum of the commercial strain. The high relative abundance of *Saccharomyces* in must differs, however, from previous observations, describing this genus as generally weakly represented (Hierro et al., 2006; Morrison‐Whittle & Goddard, 2018; Torija et al., 2001).

**Figure A4:**
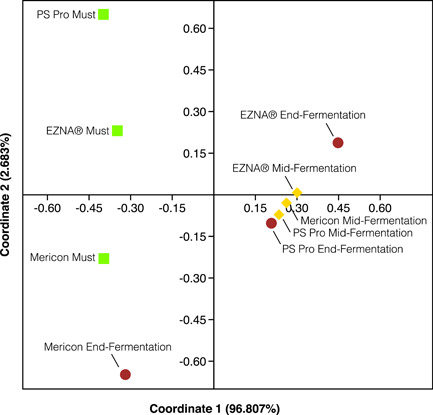
The fungal diversity of the end fermentation stage obtained via Mericon extraction method groups with must samples, rather than with other end fermentation samples. Principal coordinates analyses based on Bray–Curtis dissimilarity were computed using PAST4.

The other highly abundant taxon found in samples is *Hanseniaspora*, for which high relative abundance was observed, particularly in the mid and end fermentation stages. In must samples, sequences affiliated to *Erysiphe* sp. were identified with all kits. *Erysiphe* is a genus of a filamentous fungus, containing *E. necator*, the etiological agent of the powdery mildew. Hence, its detection in must may indicate that grapes used in the winemaking process were contaminated with this vine's pathogen. Other fungi identified in must include notably *Lachancea* and *Torulaspora*, yeasts that can both be recovered from grapes (Morata et al., 2018; Ramírez & Velázquez, 2018). Stage‐specific diversity was mostly repeatable, regardless of the kit used. One notable exception is the end fermentation sample obtained using the DNeasy Mericon extraction kit, for which the microbial community was closer to the must sample than all other stages (Figures 3 and A4). Altogether, of the three tested methods, two allowed us to obtain similar results in terms of community composition, which appear to be most impacted by the fermentation stage. The most dissimilar results, obtained using the DNeasy Mericon extraction kit (Figure A4), tend to suggest this kit underperformed for the extraction of late fermentation samples.

## 4 CONCLUSION

The present report aimed at assessing the suitability of the DNA extraction method for studying microbial diversity in the winemaking process. Among the tested extraction methods, only three yielded DNA allowing for repeatable PCR amplification of ITS amplicons. The HTS of this amplicon further suggested that two extraction methods produced repeatable and comparable results regarding PCR amplification and downstream HTS, which are E.Z.N.A® Food DNA (Omega Bio‐Tek) and DNeasy PowerSoil Pro (Qiagen). Such results might be explained by the implementation of mechanical lysis and/or optimized chemical lysis in those kits. Those methods thus seem suitable for DNA extraction and downstream applications such as HTS of wine samples at the different fermentation stages.

## AUTHOR CONTRIBUTIONS

**Antoine Gobert**: Conceptualization (lead); funding acquisition (lead); validation (equal); visualization (equal); writing—original draft (equal); writing—review and editing (equal). **Marie Sarah Evers**: Conceptualization (supporting); methodology (supporting); validation (supporting); writing—review and editing (supporting). **Christophe Morge**: Project administration (supporting); validation (supporting); writing—review and editing (supporting). **Céline Sparrow**: Project administration (supporting); validation (supporting); writing—review and editing (supporting). **Vincent Delafont**: Conceptualization (equal); data curation (lead); formal analysis (lead); methodology (equal); validation (equal); visualization (lead); writing—original draft (equal); writing—review and editing (equal).

## CONFLICT OF INTEREST

None declared.

## ETHICS STATEMENT

None required.

## Data Availability

The sequence data generated and analyzed for this study are available in the Sequence Read Archive under the project number PRJNA746771: https://www.ncbi.nlm.nih.gov/bioproject/PRJNA746771.

## 1

**Table A1:** Statistics of quality control steps performed on raw sequences obtained from a single run, using DADA2, within the QIIME2 software package

DNA extraction method	Fermentation step	Input	Filtered	Denoized	Merged	Nonchimeric
E.Z.N.A.® Food DNA Kit	End fermentation	103,950	89,538	89,411	88,674	86,569
E.Z.N.A.® Food DNA Kit	Mid fermentation	34,712	31,474	31,350	30,753	30,042
E.Z.N.A.® Food DNA Kit	Must	24,783	11,090	10,899	10,145	9235
DNeasy Mericon Food Kit	End fermentation	31,787	24,701	24,603	24,274	22,730
DNeasy Mericon Food Kit	Mid fermentation	33,865	28,422	28,310	27,808	27,018
DNeasy Mericon Food Kit	Must	85,736	44,652	44,404	42,544	42,449
DNeasy PowerSoil Pro Kit	End fermentation	33,578	28,809	28,630	27,805	26,477
DNeasy PowerSoil Pro Kit	Mid fermentation	33,139	27,212	27,080	25,856	24,912
DNeasy PowerSoil Pro Kit	Must	29,023	18,774	18,425	17,178	16,430
Total		410,573	304,672	303,112	295,037	285,862

*Note*: The final number of high‐quality sequences corresponds to the column “nonchimeric.”
